# Draft Genome Sequence of *Vibrio* sp. Strain OCN044, Isolated from Palmyra Atoll, Northern Line Islands

**DOI:** 10.1128/MRA.00042-20

**Published:** 2020-03-19

**Authors:** Rachel M. Loughran, Abigail R. Esquivel, Monica C. Deadmond, Marc J. Koyack, Brie E. Paddock, Samantha M. O’Hanlon, Blake Ushijima, Jimmy H. Saw, Patrick Videau

**Affiliations:** a Department of Biology, Southern Oregon University, Ashland, Oregon, USA; b Department of Chemistry, Southern Oregon University, Ashland, Oregon, USA; c School of Psychological Science, Oregon State University, Corvallis, Oregon, USA; d Smithsonian Marine Station, Fort Pierce, Florida, USA; e Department of Biological Sciences, George Washington University, Washington, DC, USA; Loyola University Chicago

## Abstract

*Vibrio* sp. strain OCN044 is a Gram-negative gammaproteobacterium found in marine environments. Presented here is the whole-draft genome sequence of nonpathogenic *Vibrio* sp. strain OCN044, isolated from a healthy Acropora cytherea colony off the western reef terrace of Palmyra Atoll.

## ANNOUNCEMENT

Vibrios are a group of Gram-negative bacteria found predominantly in marine and estuarine environments (1, 2). Strains of *Vibrio* spp. are implicated as etiological agents of disease for a broad range of hosts, including corals (3), mollusks (4), crustaceans (5), fish (6), and mammals (7–9). However, comparatively less is known about the mutualistic and commensal *Vibrio* strains associated with these host organisms. Strain OCN044 is a nonpathogenic constituent of the microflora associated with the reef-building coral Acropora cytherea (10), and sequencing its genome provides insight into the processes underlying such interspecies interactions.

*Vibrio* sp. strain OCN044 was isolated from a healthy *A. cytherea* colony off the western reef terrace of Palmyra Atoll in the Northern Line Islands as previously described (10). Briefly, a healthy *A. cytherea* fragment was crushed in artificial seawater, plated on glycerol seawater (GSW) medium solidified with 1.5% agar, and incubated at 29°C overnight. The isolate was purified and confirmed as a member of the *Vibrio* genus through both 16S and multilocus sequence analysis. A phenol-chloroform extraction method was used to isolate genomic DNA from an axenic culture of OCN044 grown in lysogeny broth (LB) supplemented with 3% NaCl overnight at 28°C (11). Raw reads were obtained from the Microbial Genome Sequencing Center, LLC (Pittsburgh, PA), using 151-bp paired-end read libraries, prepared as previously described (12), which were run on the Illumina NextSeq 550 platform. From high-throughput sequencing, 4,912,007 pairs of raw reads were obtained for OCN044. The raw read quality was assessed using FastQC (http://www.bioinformatics.babraham.ac.uk/projects/fastqc/), and Illumina adapter sequence removal and read quality trimming were performed using BBDuk within the BBMap package (http://sourceforge.net/projects/bbmap/) with the following parameters: ktrim=r, ordered minlen=50, mink=11, tborcomp=f, k=21, ow=t, ftm=5, zl=4, qtrim=rl, andtrimq=20.

The trimmed reads were assembled into a draft genome with SPAdes v. 3.14.0 using the “–careful” option and specifying kmers of 21, 33, 55, 77, 99, and 121 bases (13). The assembly produced 33 contigs and 30 scaffolds larger than 1,000 bases (averaging 139,795 bases and 153,784 bases, respectively) with a mean coverage of 58× and an *N*_50_ value of 666,375 bp. The draft genome sequence comprises a total of 4,623,523 bases with a 42.4% G+C content. Preliminary genome annotation of the public version conducted with the Prokaryotic Genome Annotation Pipeline (PGAP) (14) and the Rapid Annotations using Subsystem Technology (RAST) server (15) identified 4,261 and 4,365 genes, respectively. RAST analysis also indicated that, of the total genes, 1,245 nonhypothetical and 57 hypothetical genes (29% of total) were categorized into 336 subsystems. A total of 91 tRNAs were identified using tRNAscan-SE v. 2.0 (16), one of which is a predicted pseudogene.

### Data availability.

This whole-genome shotgun project has been deposited at DDBJ/ENA/GenBank under the accession number WWEU00000000. The version described in this paper is version WWEU01000000 (GCF_009857245.1). The raw sequence reads were deposited in the SRA under accession number SRR11002660 and are associated with BioSample number SAMN13735740.
